# Binding and Synergizing Motif within Coleopteran Cadherin Enhances Cry3Bb Toxicity on the Colorado Potato Beetle and the Lesser Mealworm

**DOI:** 10.3390/toxins11070386

**Published:** 2019-07-02

**Authors:** Youngjin Park, Gang Hua, Suresh Ambati, Milton Taylor, Michael J. Adang

**Affiliations:** 1 Department of Entomology, University of Georgia, Athens, GA 30602, USA; 2 Department of Genetics, University of Georgia, Athens, GA 30602, USA; 3 Department of Biochemistry and Molecular Biology, University of Georgia, Athens, GA 30602, USA

**Keywords:** Cry, *Bacillus thuringiensis*, cadherin, Cry3b, Coleoptera, insecticidal toxin

## Abstract

Cry3Bb toxin from *Bacillus thuringiensis* is an important insecticidal protein due to its potency against coleopteran pests, especially rootworms. Cadherin, a protein in the insect midgut epithelium, is a receptor of Cry toxins; in some insect species toxin-binding domains of cadherins-synergized Cry toxicity. Previously, we reported that the DvCad1-CR8-10 fragment of *Diabrotica virgifera virgifera* cadherin-like protein (GenBank Accession #EF531715) enhanced Cry3Bb toxicity to the Colorado Potato Beetle (CPB), *Leptinotarsa* *decimlineata* (*L. decimlineata*). We report that individual CR domains of the DvCad1-CR8-10 fragment were found to have strong binding affinities to α-chymotrypsin-treated Cry3Bb. The dissociation constant (*K_d_*) of Cry3Bb binding to the CR8, CR9, and CR10 domain was 4.9 nM, 28.2 nM, and 4.6 nM, respectively. CR8 and CR10, but not CR9, enhanced Cry3Bb toxicity against *L. decimlineata* and the lesser mealworm *Alphitobius diaperinus* neonates. In-frame deletions of the DvCad1-CR10 open reading frame defined a high-affinity binding and synergistic site to a motif in residues I1226–D1278. A 26 amino acid peptide from the high affinity Cry3Bb-binding region of CR10 functioned as a Cry3Bb synergist against coleopteran larvae.

## 1. Introduction

The Cry3 insecticidal proteins of *Bacillus thuringiensis* (Bt) are toxic to larvae of beetles (Order: Coleoptera) that are some of the most important agricultural pests worldwide. The widest usage of Cry3 proteins is found in corn via in planta expression of Cry3Aa and Cry3Bb for rootworm management. The Cry3Aa protein was also shown to be produced in genetically modified potatoes for the control of the Colorado potato beetle (CPB) [1,2]. CPB is a serious pest of potato, due to development of resistance to many chemical insecticides. Alyokhin et al. (2007) [3] reported increasing resistance in CPB against neonicotinoids, a chemical class that had been effective in controlling CPB populations. Novodor^TM^, a Bt biopesticide containing Cry3Aa crystal proteins and spores, is active against CPB and is reportedly effective against the first and second larval stages but not in the third instar and later stages [4,5]. These limitations to Bt control of CPB are driving factors in the development of a Bt synergist.

Cadherin proteins have domains called cadherin repeats (CR) that are flanked by structural elements, which together functionally enable cell adhesion, cytoskeletal organization, and morphogenesis. In insects, cadherins received attention for their roles in Bt Cry action and acquired insect resistance (reviewed in [6,7]). With respect to Coleoptera, cadherins have inherent physiological roles while functioning as adventitious receptors of Cry3 toxins in some insect species, including mealworms *Tenebrio molitor*, *Tribolium castaneum*, and *Alphitobius diaperinius* [8,9,10]. After insects ingest Cry proteins, the activated Cry toxin binds cadherins tethered to the external side of midgut membranes, with high affinity, then the toxin oligomerizes and inserts into the membrane. A surprising aspect of Cry- cadherin interactions is that peptide fragments of cadherins containing binding sites can synergize Cry toxicity to insects [11]. In some cases, the cadherin fragment induces toxin oligomerization, replacing the midgut cadherin binding step [8,12,13].

Park et al. (2009) [14] identified a cadherin fragment CR8-10 from western corn rootworm (WCR), *Diabrotica virgifera virgifera* cadherin [15], as a synergist of Cry3Aa and Cry3Bb toxicity. The DvCad1-CR8-10 fragment bound Cry3Aa and Cry3Bb with a high nanomolar affinity and enhanced Cry3 toxicity to the larvae of the Colorado potato beetle (CPB), *Leptinotarsa decemlineata* (Coleoptera: Chrysomelidae), and the lesser mealworm (LMW), *A. diaperinius* (Coleoptera: *Tenebrionidae*) [14]. Recent evidence [16] showed that DvCad1 is probably not a receptor of Cry3Aa as knock-down of DvCad1 by RNA inhibition (RNAi) did not reduce Cry3Aa toxicity.

In this study, we investigated the location of Cry3Bb sites in DvCad1-CR8-10 and demonstrated that CR8, CR9, and CR10 each had a high-affinity Cry3Bb binding site and that CR8 and CR10 enhanced Cry3Bb toxicity against CPB and LMW. Each Cry3Bb binding site located in CR8-10 was bound by a common site on Cry3Bb. A 26 amino acid region of DvCad1-CR10 was identified as critical for Cry3Bb binding and toxicity enhancement against CPB and LMW.

## 2. Results

### 2.1. Construction of Individual CR Regions for DvCad1-CR8-10 and Testing Enhancement of Cry3Bb Toxicity to CPB and LMW

**Figure 1:**
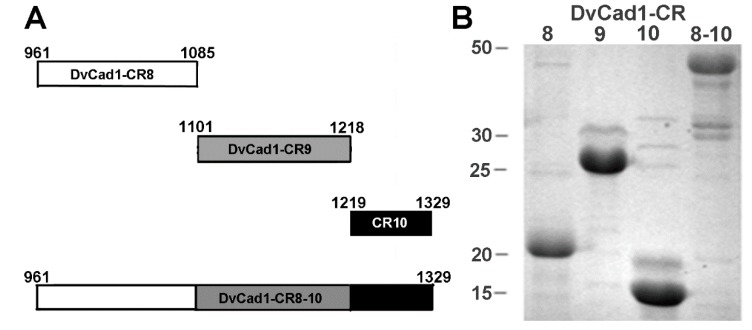
DvCad1-CR8, CR9, CR10, and CR8-10 regions of *Diabrotica virgifera virgifera* midgut cadherin (**A**), were expressed in *Escherichia coli* and the inclusion peptides, separated by 12% SDS-PAGE (**B**).

The CR8-10 region of WCR cadherin [15] bound Cry3Aa and Cry3Bb toxins with high affinity, and synergized toxicity to the coleopteran larvae [14]. Figure 1A shows the CR constructs made to identify the regions of CR8-10 peptide involved in the binding and synergy of Cry3 toxins. We individually expressed the coding regions of CR8 (14-kDa), CR9 (13-kDa), and CR10 (13-kDa), and CR8-10 (45-kDa) in *Escherichia coli* (*E. coli*). Inclusions isolated from *E. coli*-expressing CR regions were separated by SDS-PAGE (Figure 1B). While the CR10 and CR8-10 peptides sizes were as expected; CR8 and CR9 migrated as 20- and 25-kDa peptides, and the apparent molecular sizes were larger than expected.

### 2.2. Testing CR8, CR9, and CR10 for Enhancement of Cry3Bb Toxicity to CPB and LMW

**Figure 2:**
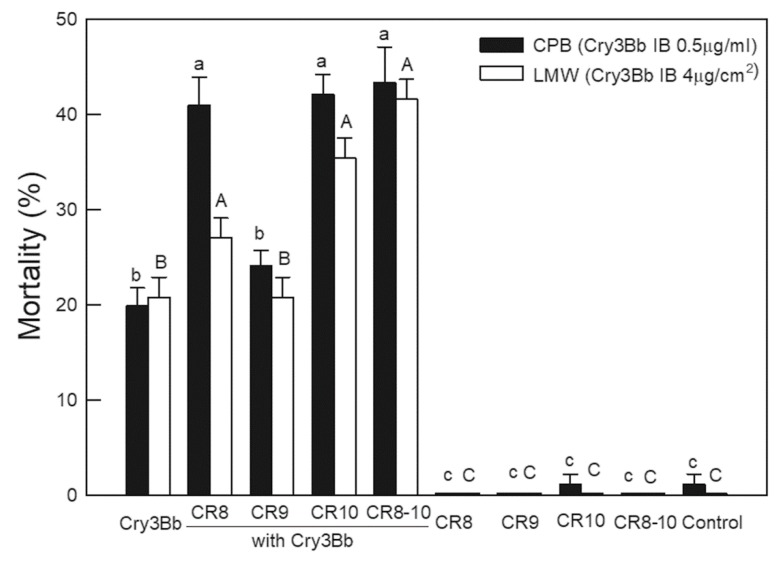
Bioassays of the DvCad1-CR8, CR9, CR10, or CR8-10 inclusions with Cry3Bb against the Colorado potato beetle (CPB) and the lesser mealworm (LMW). Suspensions of Cry3Bb inclusions (0.5 µg/mL), with or without CR inclusions (5.0 μg/mL), at a toxin/peptide mass ratio of 1:10, were sprayed onto the excised potato leaves. Suspensions of Cry3Bb inclusions (4 μg/cm^2^), with or without single DvCad1-CR inclusions (40 μg/cm^2^), at a toxin/peptide mass ratio of 1:10 were fed to the LMW larvae in a surface overlay bioassay. Larval mortality was scored on day 3. Points annotated with the same letter were not significantly different. Lowercase letters compare CPB, while uppercase letters compare LMW.

DvCad1-CR8 and -CR10 fragments enhanced Cry3Bb toxicity to CPB and LMW larvae (Figure 2). Leaflets of potato were sprayed with suspensions of Cry3Bb crystals alone or with DvCad1-CR inclusions, and were fed to the first instar CPB larvae. The concentration of Cry3Bb crystals on the potato leaves caused about 20% mortality against the first instar CPB larvae. The addition of DvCad1-CR8, -CR10, and CR8-10 inclusions at a 1:10 (Cry3Bb:DvCad1-CR peptide) mass ratio to Cry3Bb, each, increased larval mortality to 41.0 ± 2.9%, 42.1 ± 2.1%, and 43.3 ± 3.8%, respectively. The mortality of the CPB larvae-fed Cry3Bb plus DvCad1-CR9 at 23.1 ± 1.6% was not significantly different from Cry3Bb alone.

We tested the addition of DvCad1 CR peptides on the Cry3Bb-induced mortality to LMW larvae, using 4 μg Cry3Bb/cm^2^ diet surface that caused 20% larval mortality. Combinations of CR8, CR10, and CR8-10 inclusions with Cry3Bb at a 1:10 (Cry3Bb:CR peptide) mass ratio, increased larval mortality to 27.1 ± 2.2%, 35.4 ± 4.2%, and 41.6 ± 5.2%, respectively. Similar to the results of the CPB bioassays, CR9 peptide did not increase LMW mortality, relative to the Cry3Bb alone. DvCad1-CR peptides alone were not toxic to the first instar CPB larvae or LMW. Peptide CR10, of the three individual CR units, was the most active synergist of the Cry3Bb in the CPB and LMW larval bioassays.

### 2.3. CR8, CR9, and CR10 Peptides Bound Cry3Bb and Competed for the Same Binding Site on Cry3Bb

**Figure 3:**
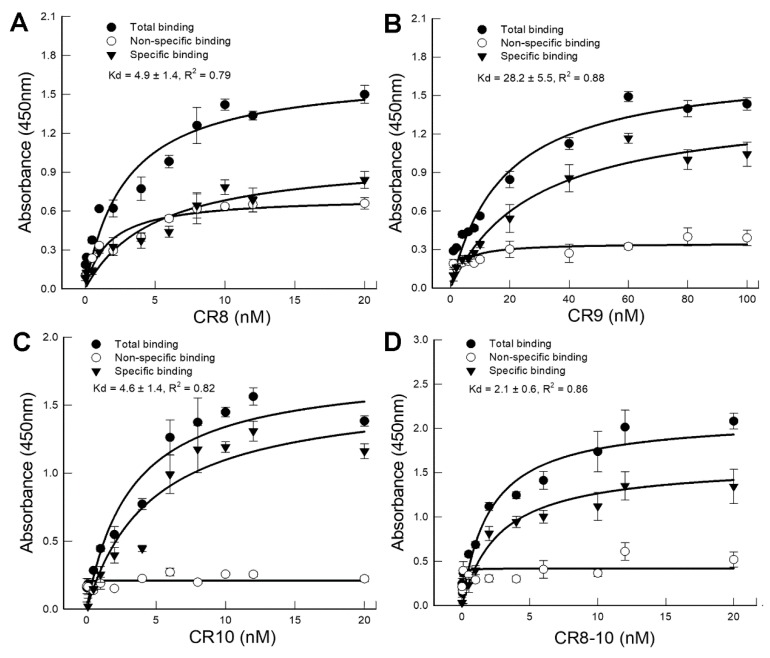
Binding of DvCad1-CR8 (**A**), DvCad1-CR9 (**B**), DvCad1-CR10 (**C**), and DvCad1-CR8-10 (**D**) peptide to Cry3Bb. Microtiter plates coated with 1.0 μg of chymotrypsinized Cry3Bb were incubated with increasing concentrations of biotinylated DvCad1-CR peptide alone or with a 1000-fold molar excess of unlabeled homologous CR peptide, to determine specific binding. Each data point represent the mean of the results from two experiments, done in duplicates. Error bars depict the standard deviations. Binding affinities (*K_d_*) were calculated on the basis of specifically bound biotinylated DvCad1-CR peptide, using a one-site saturation binding equation.

Binding of DvCad1 CR8-10 peptides to Cry3Bb toxin was analyzed by an ELISA protein binding assay (Figure 3). Chymotrypsinized-Cry3Bb was 55-kDa (data not shown), as expected [17]. Biotin-labeled DvCad1 CR peptides were bound Cry3Bb specifically, saturably, and with high affinity (CR8 *K_d_* = 4.9 ± 1.4 nM; CR9 *K_d_* = 28.2 ± 5.5 nM; CR10 *K_d_* = 4.6 ± 1.4 nM; and CR8-10 *K_d_* = 2.1 ± 0.6 nM). The CR9 peptide, with the lowest Cry3Bb binding affinity of the CR8 to 10 peptides was also the peptide without Cry3Bb synergy.

**Figure 4:**
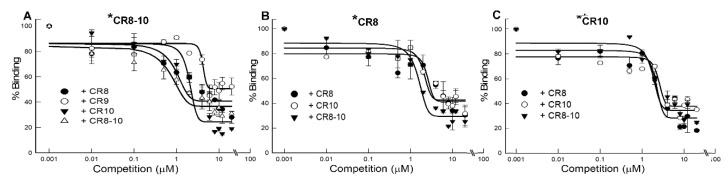
Competition binding of DvCad1-CR8-10 (**A**), CR8 (**B**), and CR10 (**C**) to Cry3Bb. Microtiter plates coated with Cry3Bb were incubated with 10 nM of biotin-DvCad1 fragment and increasing concentration of unlabeled DvCad1-CR8-10, CR8, or CR10.

Competition for a shared CR binding site on Cry3Bb was analyzed by measuring the labeled-CR fragment binding to Cry3Bb in the presence of increasing concentrations of unlabeled CR fragment. In Figure 4A, biotinylated-DvCad1-CR8-10 (10 nM) was competed by increasing the amounts of unlabeled CR8, CR9, CR10, or CR8-10. Binding of biotin DvCad1-CR8-10 to Cry3Bb was displaced by increasing concentrations of the unlabeled CR8, CR10, and CR8-10 peptide, which evidenced that CR8 and CR10 acted interchangeably in reducing CR8-10 binding to Cry3Bb. A higher concentration of CR9 was required before a reduction of biotin-DvCad1-CR8-10 binding was measured and the extent of competition was found to be less than that with CR8, CR10, or CR8-10 peptides. To further test the similarity of interactions between CR8 and CR10 peptides with Cry3Bb, we biotinylated CR8 and CR10, and measured the capacity of increasing amounts of CR8, CR10, and CR8-10, to compete binding to Cry3Bb. As shown in Figure 4B,C, the competition curves were similar for biotin-DvCad1-CR8 and biotin-DvCad1-CR10. These competition results agreed with the saturation binding results, revealing similar affinities for the CR8 and CR10 peptide binding to Cry3Bb, and additionally suggest that they bind the same site(s) on Cry3Bb.

### 2.4. The N-Terminal Third of DvCad1-CR10 Binds Cry3Bb and Enhances Cry3Bb Toxicity to Coleopteran Larvae

**Figure 5:**
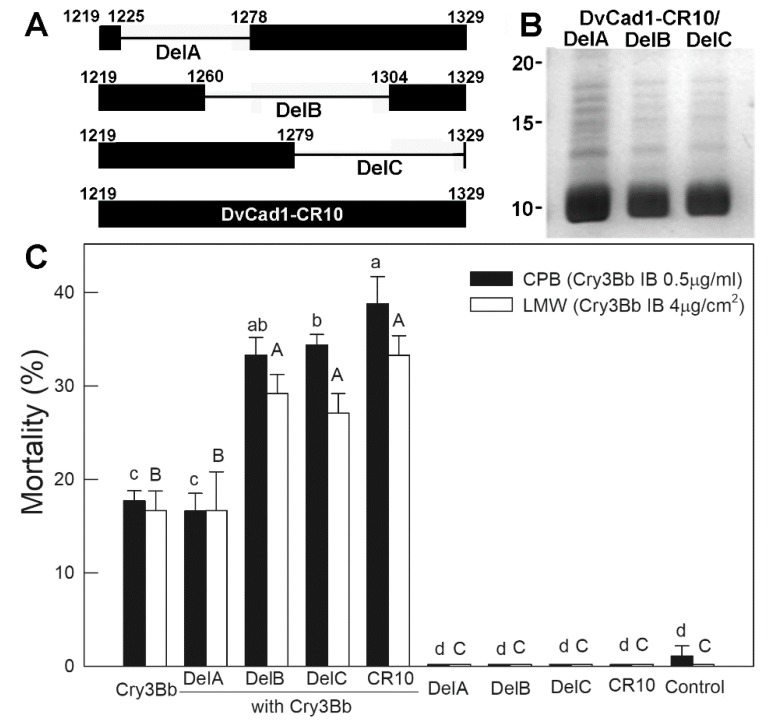
Diagram of DvCad1-CR10 deletion constructs (**A**), peptides separated by SDS-PAGE (**B**), and bioassays with Cry3Bb and CR10 deletion peptides (**C**). Details of the bioassays were as in Figure 1. Larval mortality was scored on day 3. Points annotated with the same letter were not significantly different. Lowercase letters compared the CPB, while the uppercase letters compared the LMW.

We removed the overlapping regions of DvCad1-CR10 by in-frame deletion to construct CR10/DelA, CR10/DelB, and CR10/DelC (Figure 5A). Each construct was expressed in *E. coli* as an inclusion made of 10-kDa CR10/Del peptide (Figure 5B). Cry3Bb was fed to CPB larvae alone or with 1:10 mass ratio of CR10/Del peptide and mortality was scored after 3 days. Peptide DvCad1-CR10 increased larval mortality from 17.7 ± 1.1% to 38.8 ± 2.8%, and peptides CR10/DelB and CR10/DelC increased mortality to 33.3 ± 1.9% and 34.4 ± 1.1%, respectively (Figure 5C); in contrast CR10/DelA inclusions did not increase Cry3Bb toxicity to CPB or LMW.

**Figure 6:**
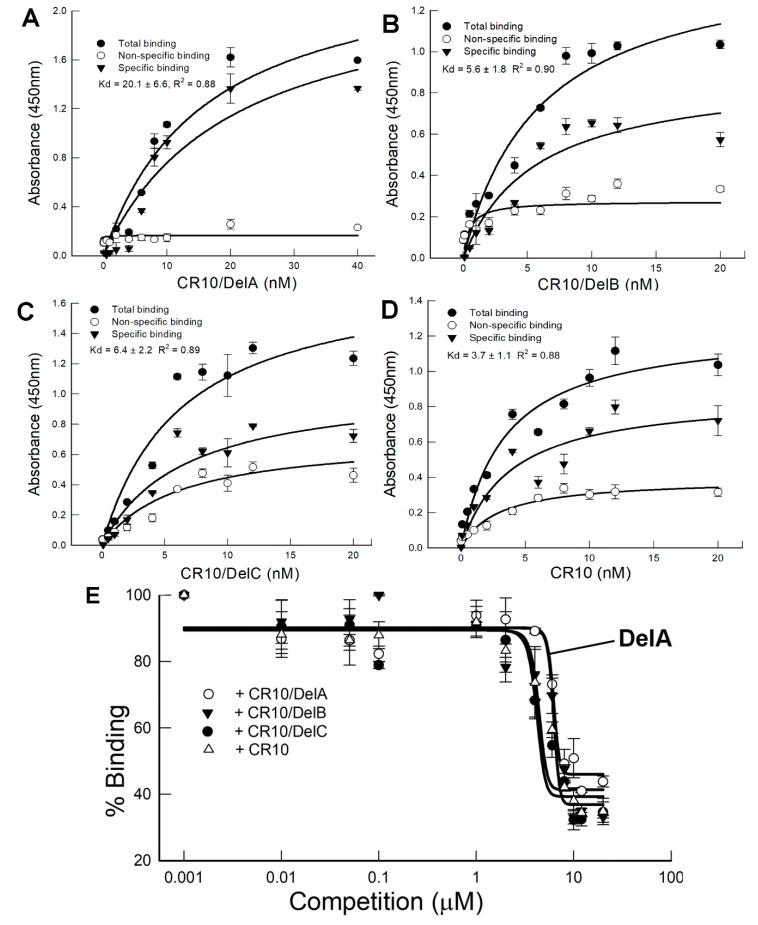
Binding of the biotinylated DvCad1-CR10/DelA (**A**), CR10/DelB (**B**), CR10/DelC (**C**), and CR10 (**D**) to Cry3Bb was determined by an ELISA binding assay. Microtiter plates coated with Cry3Bb were incubated with increasing concentrations of biotinylated CR10 peptide alone or with a 1000-fold molar excess of the unlabeled homologous peptide, to determine specific binding of Cry3Bb. (**E**) shows binding of biotin-CR10 to Cry3Bb, with increasing concentrations of CR10 or CR10/Del peptides.

The binding properties of CR10 deletion peptides were compared with CR10 in saturation and competition binding assays (Figure 6). While each peptide bound saturably with calculated *K_d_* affinities for DvCad1-CR10/DelA, -CR10/DelB, and -CR10/DelC peptides for Cry3Bb of 20.6 ± 1.8 nM, 6.4 ± 2.2 nM, and 5.6 ± 1.8 nM, respectively (Figure 6A–C), the lower affinity of CR10/DelA was consistent with a lack of Cry3Bb enhancement. In competition binding assays, biotin-DvCad1-CR10 binding to Cry3Bb was reduced by increasing the concentrations of the unlabeled DvCad1-CR10, CR10/DelA, CR10/DelB, and CR10/DelC (Figure 6E). More CR10/DelA was required before biotin-DvCad1-CR10 binding was reduced, a result consistent with a reduced binding affinity to Cry3Bb.

### 2.5. A 26-Amino Acid (1254I - S1279) Peptide from Region A of CR10 Enhances Cry3Bb Toxicity

**Figure 7:**
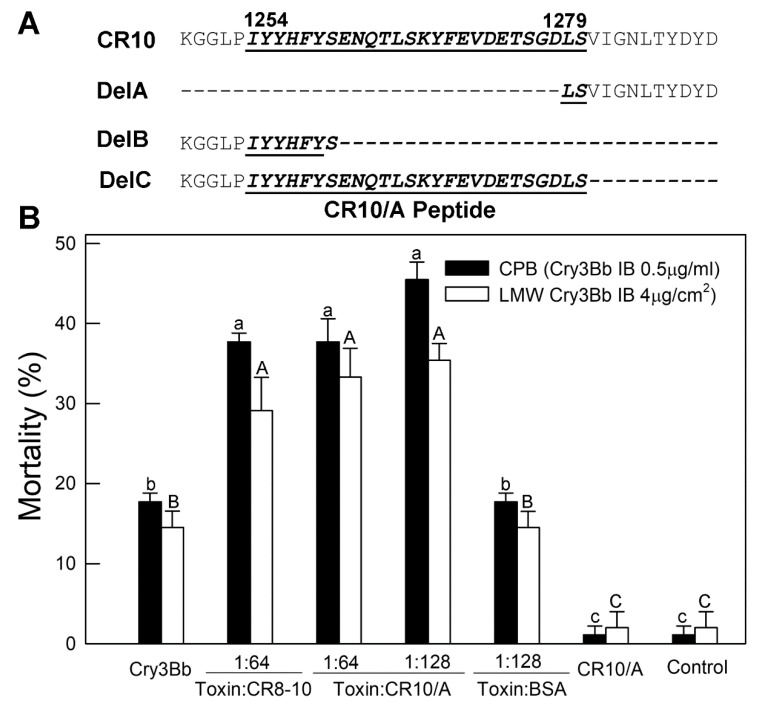
The CR10/A peptide enhances Cry3Bb toxicity to CPB and LMW neonate larvae. (**A**) Residues of DvCad1-CR10 aligned with CR10 in-frame deletions and CR10/A peptide. Dashes depict amino acids absent in the expressed Del constructs. (**B**) Bioassay results where suspensions of the Cry3Bb crystals were sprayed on the excised potato leaves or applied to the LMW diet alone or with CR8-10 peptide or soluble CR10/A peptide. A Cry3Bb:CR10/A molar ratio of 1:64 was equivalent to a 1:2.7 mass ratio. Larval mortality was scored on day 3. BSA and the CR10/DelA peptide were not toxic to larvae.

Reduced Cry3Bb binding and a lack of toxicity enhancement by CR10/DelA, identified 51-aa residues deleted from fragment CR10 as being important to the Cry3Bb interactions. Figure 7A shows the synthetic 26-aa residue CR10/A peptide used to directly test the toxicity enhancement function for this small region of CR10. Fragments CR10/DelB and DelC, which contained the overlapping segments of the CR10/A region and retained a high affinity binding and synergy of Cry3Bb toxicity, were also considered in the design of the CR10/A (Figure 7A). Synthetic CR10/A peptide was tested with an amount of Cry3Bb that alone caused about 20% mortality to CPB and LMW. As shown in Figure 7, CR10/A peptide at a 1:64 (toxin:peptide) molar ratio, enhanced Cry3Bb toxicity to both CPB and LMW, and similarly to CR8-10. The increased synergistic effect at a 1:128 (toxin:peptide) molar ratio was not statistically significant. The control bioassay using the BSA showed no synergistic effect with Cry3Bb.

## 3. Discussion

This study identified multiple sites on DvCad1-CR8-10 involved in Cry3Bb high-affinity binding and synergy [14]. While CR8, CR9, and CR10 each bound Cry3Bb toxin with high affinity; the CR8 and CR10 regions with binding constants of *K_d_* = 5 nM synergized Cry3Bb toxicity to CPB and LMW, but CR9 peptide with a *K_d_* = 28 nM did not. The *E. coli* expressed peptides CR8 and CR9 had apparent molecular sizes on the SDS-PAGE that were larger than expected. Particularly for CR9, we did not know if the two-times difference between the apparent and calculated sizes for the peptide, affected the Cry3Bb binding or synergy. Multiple Cry toxin binding sites occurred on different CR regions of an insect’s midgut cadherins. For example, lepidopteran active Cry1Ab recognized three binding sites on *Manduca sexta* cadherin, with the binding site in the CR region nearest to the plasma membrane being considered to be the most important to Cry1Ab toxicity [18].

Fragment CR10 of DvCad1 contained multiple Cry3Bb binding sites. Region A of CR10 (residues 1227–1277) contained a site that bound Cry3Bb with a *K_d_* of 5 nM and regions B and C had a binding site with a *K_d_* = 20 nM. Fragments CR10/DelA and CR9 bound Cry3Bb at *K_d_* = 20 nM and 28 nM and both failed to synergize toxicity; other DvCad1 fragments bound Cry3B at *K_d_* = 5 nM and synergized higher toxicity. These results suggest that seemingly small differences in the affinities of the cadherin fragment–Cry interaction, impact the synergistic effects of a cadherin fragment.

Three domain Cry toxins bind patterns of amino acids via domain 2 loops, based on inverted hydropathic complementarity [19]. Correspondingly, insect cadherins have residue motifs with hydropathic complementarity matching to the Cry domain 2 loops. Cry3Aa binding sites are identified in *Tribolium castaneum* and *Tenebrio molitor*, and a Cry3Bb binding site was predicted in *A. diaperinus* cadherin [8,9,10]. There was no obvious Cry3 binding motif within the CR10/A 26-residue peptide to fit into the complementarity pattern; the peptide did have four residues which overlapped a predicted Cry3 binding motif (1277GDLSVIGN1284). However, fragment CR10/DelB which binds and synergizes Cry3Bb, did not have these aa residues, a result which argued against 1277GDLS1280 as being involved in toxin interaction. Seven aa residues (1254IYYFYS1260) of synthetic CR10/A peptide overlapped CR10/DelB and CR10/DelB, suggesting that those 7 aa residues could be involved in Cry3Bb interactions. Additional experimentation is needed to identify the specific Cry3Bb binding motif in CR10/A peptide.

How do cadherin fragments synergize three-domain Cry protein toxicity to insect larvae? Cry toxins form oligomers, in vitro, upon contact with synergistic cadherin fragments [12]. The ability of a cadherin fragment to augment a step in Cry action, such as oligomerization, is consistent with the ability of a cadherin fragment to restore Cry3Bb susceptibility after a cadherin loss knockdown by RNAi [10]. It is interesting that DvCad1 fragments enhanced Cry toxicity against WCR [14], even if DvCad1 was probably not a Cry receptor in [16]. Similarly, cadherin might not be a receptor in *L. decimlineata* as ADAM10 metalloprotease is the only identified Cry3 receptor in that species [20]. The implication is that Cry contact with a specific type of binding motif is a key step in three-domain Cry toxin action. Such a functional step could occur in vivo upon contact with a cadherin receptor, a similar motif on sodium solute receptor, or when the cadherin fragment is fed per oss with Cry toxin. DvCad1 is probably not a Cry3 receptor in WCR as knock-down of DvCad1 expression did not reduce Cry3Aa susceptibility [16]. The ability of DvCad1 fragments and CR10/A peptide to enhance Cry3Bb toxicity suggests that synergistic cadherin fragments do augment a step in three-domain Cry toxin action.

## 4. Conclusions

Our research focused on the Cry3Bb-binding and toxicity regions of DvCad1-CR8-10. We identified CR8 and CR10 fragments as having high nM Cry3Bb-binding and toxicity-enhancement properties. Deletion analyses of CR10 identified a region involved in Cry3Bb interactions, and 26-aa CR10/A peptide was sufficient for both binding and toxicity enhancement. Further research of the interaction between Cry3 toxins and DvCad1 could help to better understand the insecticidal mechanism of Bt toxin and the phenomenon of toxicity enhancement by cadherin and could lead us to open a new prospect in the field of pest control.

## 5. Materials and Methods

### 5.1. Preparation of Cry3Bb Toxin and DvCad1 CR Peptides

The construction and expression of Cry3Bb and DvCad1-CR8-10 peptide in the *E. coli* strain BL21-CodonPlus (DE3/pRIL (Stratagene, La Jolla, CA, USA) are published in [14]. Over-expressed Cry protein or cadherin peptides were deposited in the inclusion bodies and released from the harvested *E. coli*, by treatment with lysis buffer (50 mM Tris, 10 mM EDTA, 15% sucrose, plus lysozyme). Inclusion bodies were harvested by centrifugation, washed, and stored, as described in [14]. Total protein was measured by a Bio-Rad protein assay using bovine serum albumin (BSA) as the standard. For toxin-cadherin microplate binding assays, Cry3Bb was solubilized from the washed inclusions in 50 mM NaHCO_3_, activated by chymotrypsin, and dialyzed against 50 mM NaHCO_3_ overnight at 4 °C, according to [21]. Cadherin peptide inclusions were solubilized in 8 M urea with 20 mM Na_2_CO_3_ (pH 9.6) and the peptide was purified on a HiTrap Ni^2+^-chelating HP column (GE Healthcare, Piscataway, NJ), according to [11], and then dialyzed against the phosphate buffer saline at 4 °C, overnight. The total protein was measured by the Bio-Rad (Hercules, CA, USA) protein assay, using bovine serum albumin (BSA) as a standard. The purity of the Cry3Bb toxin and the cadherin peptide was assessed by 12% sodium dodecyl sulfate-polyacrylamide gel electrophoresis (SDS-PAGE) with Coomassie blue R-250 staining and gel image analysis (Alpha Innotech, San Leandro, CA, USA), with BSA as a standard.

### 5.2. Cloning Individual DvCad1-CR8, -CR9, or -CR10 Peptides from the DvCad1-CR8-10 Peptide

Specific primers for amplifying and sub-cloning DvCad1-CR8 (^961^V-I^1085^) and -CR10 (^1219^Q-W^1329^) fragments from pET-30/DvCad1-CR8-10 [14] are listed in Appendix A. Polymerase chain reaction (PCR) amplifications were performed using the Expand High Fidelity PCR System kit (Roche Diagnostics, Indianapolis, IN) with pET-30/DvCad1-CR8-10 as a template, through 25 cycles of 94 °C for 30 s, 55 °C for 30 s, and 68 °C for 1 min. The strategy for deleting a single fragment from DvCad1-CR8-10 was based on the Stratagene Quick Change method, as described in [11]. Briefly, 5’- and 3’- primer pairs containing terminal EcoRI cleavage sites were designed, complementary to the region to be deleted. The amplicon was gel purified (Qiagen) and digested with DpnI and EcoRI, ligated, and the DNA product transformed into *E. coli* BL21. The coding regions of all deleted plasmids were sequenced. The DvCad1-CR9 (^1101^Q-F^1218^) fragment was cloned by PCR, using the pET-30/DvCad1-CR8-10 plasmid as a template with CR9/FWD, which had an EcoRI site, and CR9/REV, which had a HindIII site. The PCR product was inserted between the EcoRI and HindIII cleavage sites of plasmid pET-30a (+) (Novagen, Madison, WI) and transformed into the *E. coli* BL21 after ligation. The coding sequences were confirmed through sequencing (Georgia Genomics Facility, University of Georgia, USA).

### 5.3. Deletions of the Cry3Bb-Binding Epitopes in DvCad1-CR10

To identify a region of DvCad1-CR10 that is critical for Cry3Bb-binding and enhancement of Cry3Bb toxicity, DvCad1-CR10/DelA, -CR10/DelB, and -CR10/DelC were constructed, with the respective clones having a region of CR10 deleted from the N-terminal (^1226^S-D^1277^), middle (^1261^E-Q^1303^), or C-terminal (^1280^V-W^1329^) region. This was accomplished using an in-frame deletion method, as described in [11], and pairs of primers with EcoRI cleavage sites at their ends (Table A1). PCR amplifications were performed with pET-30/DvCad1-CR10 as template, through 30 cycles of 94 °C for 30 s, 52 °C for 30 s, and 72 °C for 1 min. Cloning, expression, and peptide purification protocols for the DvCad1-CR10 deletion inclusions were as described above, for the single DvCad1-CR fragments.

### 5.4. Microplate Assays for Measuring DvCad1-CR Fragment Binding to Cry3Bb Toxin

We used a microplate enzyme-linked immunosorbent assay (ELISA) for measuring protein-protein interactions [22], as described in [14]. Purified DvCad1-CR peptides were biotinylated using sulfo-NHS (N-hydroxysuccinimide)-photocleavable biotin (Pierce, Rockford, IL, USA), dialyzed into 200 mM NaCl, 20 mM Na_2_CO_3_ (pH 8.0), and stored in aliquots at 4 °C. Microtiter plates (high-binding, 96-well Immulon 2HB plates; Thermo Fisher Scientific, Inc., Waltham, MA) were coated with 1.0 µg Cry3Bb/well in 50 μL coating buffer (100 mM Na_2_CO_3_, pH 9.6), with or without the 1000-fold molar excess unlabeled DvCad1-CR peptides. In competition binding assays, 20 nM biotinylated DvCad1-CR peptide was mixed with increasing concentrations of the unlabeled DvCad1-CR peptides. Cry3Bb toxin-coated or peptide-coated plates were washed and incubated with horse radish peroxidase-conjugated streptavidin (SA-HRP; Pierce), and then incubated with an HRP chromogenic substrate (1-Step Ultra TMB-ELISA, Thermo Fisher Scientific, Inc., USA), after washing, to detect bound SA-HRP. Color development was stopped by adding 3M sulfuric acid, and absorbance was measured at 450 nm using a microplate reader (MDS Analytical Technologies, Sunnyvale, CA, USA). Data were plotted and analyzed using the Sigma Plot Version 11.0 (SPSS Science, Chicago, IL, USA).

### 5.5. Insect Bioassays

A colony of CPBs founded from adults provided by the New Jersey Department of Agriculture (Trenton, NJ) was maintained on potato plants, and larval bioassays were conducted according to [14]. Suspensions (30 ml) of Cry3Bb inclusions with or without DvCad1-CR fragment inclusions were prepared in diluent (0.12% vol/vol Kinetic (Helena Chemical Co. Collierville, TN, USA) plus 0.12% vol/vol spreader-sticker (polyethylene glycol and octyl phenol polyethoxy ethanol; Southern Agricultural Insecticides, Inc., Boone NC, USA) in tap water). To study the synergistic effects of DvCad1 peptides in bioassays against CPB, aliquots of 5.0 µg/mL of DvCad1-CR peptide inclusions were added to 0.5 μg/mL of Cry3Bb inclusions, to yield a final 1:10 (Cry3Bb:DvCad1-CR) mass ratio suspension. Suspensions were sprayed on the potato leaves, which were then air-dried in a fume hood for 15 min. Each bioassay was conducted with 30 larvae per replicate and three replicates per treatment. Control bioassays were performed using BSA that did not enhance the toxicity of the Cry3Bb toxin.

A colony of LMWs was founded from the adults, provided by Dr. Nancy Hinkle (University of Georgia). The colony was maintained on containerized pine shavings, chicken feed (Purina Mills LLC, St. Louis, MO, USA), and apple slices, at 28 °C and 70% relative humidity, with a photoperiod of 16 h light and 8 h darkness [10]. Surface bioassays were conducted on a semi-solid chicken feed diet, as previously described [10]. Cry3Bb inclusions alone or with DvCad inclusions were serially diluted with sterile deionized water and then overlaid onto the diet surface and air-dried. One larva was transferred into each well; the trays were sealed with perforated lids (C-D International, Pitman, NJ, USA) and then covered with brown paper, to provide a dark environment until mortality was assessed.

### 5.6. Insect Bioassays with DvCad1 N-Terminal Region (1254T - S1279) Peptide and Cry3Bb

The 26 amino acid residue peptide (IYYHFYSENQTLSKYFEVDETSGDLS) corresponding to a N-terminal region of DvCad1-CR10 was synthesized by United Peptide Corporation (Rockville, MD, USA) and used with Cry3Bb in insect bioassays. The synthetic peptide was dissolved in distilled water and stored at 4 °C, until used. The synergistic effect of the synthetic peptide was compared with CR8-10 peptide, using Cry3Bb at 0.5 μg/mL and a 1:64 molar ratio of CR8-10 or CR10/A synthetic peptide.

### 5.7. Statistical Analysis

Mortality data from the bioassays were normalized using arcsine-square root (χ) transformation and were analyzed using analysis of variance (ANOVA), with the significance level set at an α value of 0.05. When significant *F* values were detected, the means were separated using a Fisher protected least significant difference (LSD) test, to compare the treatment means with the control and with each other. All calculations were performed using PROC GLM and PROC UNIVARIATE of the Statistical Analysis System (SAS 2002–2003, Version 9.1; SAS Institute, Cary, NC, USA). All data are presented in the original scale.

## Appendix A.

**Table A1:** Nucleotide primers used in this study to construction of a single cadherin fragments from a DvCad1-CR8-10 and cloning of DvCad1-CR10 deletions from the DvCad1-CR10 fragment.

Primer	Orientation	Primer DNA Sequence
Primers for construction of single cadherin fragments (DvCad1-CR8, -CR9, or -CR10)		
DvCad1-CR8-F	Forward	5’-CCCAgaattcCACCACCACCACCACCACTAAA-3’
DvCad1-CR8-R	Reverse	5’-CTGGgaattcAATGCTAGGGGTCTGGTAGTTCC-3’
DvCad1-CR9-F	Forward	5’-CCCAgaattcATTTTCCCAGAGAACGACCAGA-3’
DvCad1-CR9-R	Reverse	5’-CAGAaagcttAAAGATTGGCTGAGCATCGTTGTT-3’
DvCad1-CR10-F	Forward	5’-GCCAgaattcCAGAACTCTGACTGGTCTGTTTC-3’
DvCad1-CR10-R	Reverse	5’-TAAGgaattcCATATGTATATCTCCTTCTTAAAGTTAA-3’
Primers for cloning of DvCad1-CR10 deletions (DvCad1-CR10/DelA, -CR10/DelB, or -CR10/DelC)		
DvCad1-CR10/DelA-F	Forward	5’-TTCTgaattcCTGTCTGTTATTGGTAACCTGAC-3’
DvCad1-CR10/DelA-R	Reverse	5’-CAACgaattcAACAGACCAGTCAGAGTTCTG-3’
DvCad1-CR10/DelB-F	Forward	5’-CGATgaattcGTACGCATGCTGGACCCA-3’
DvCad1-CR10/DelB-R	Reverse	5’-TCTGgaattcAGAGTAGAAATGGTAATAGAT-3’
DvCad1-CR10/DelC-F	Forward	5’-CCCAgaattcCACCACCACCACCACCACTAAA-3’
DvCad1-CR10/DelC-R	Reverse	5’-TACCgaattcAGACAGGTCACCAGAAGTCTC-3’

^a^ Endonuclease cleavage sites are italicized.
